# Respiratory Viruses, Self-Diagnosis, Early Treatment and Prophylaxis

**DOI:** 10.3390/v18060650

**Published:** 2026-06-04

**Authors:** Anna Puigdellívol-Sánchez, Celia Lozano-Paz, Roger Valls-Foix, Ignacio Morán-Blanco, Ignasi Calicó-Bosch, Mariana Castells

**Affiliations:** 1 Primary Health Care, CAP Anton de Borja-Centre Universitari, Consorci Sanitari de Terrassa (CST), c/Marconi-Cantonada Edison s/n, 08191 Rubí, Spain; 2 Human Anatomy and Embryology Unit, Faculty of Medicine and Health Sciences, Universitat de Barcelona, Casanova 143, 08036 Barcelona, Spain; 3 Primary Health Care, Centro de Salud Yepes, Servicio de Salud de Castilla-La Mancha (SESCAM), Av Sta Reliquia 26, 45313 Toledo, Spain; 4 Laboratory of Virological Diagnosis, Hospital Vall d’Hebron (Barcelona), Passeig de la Vall d’Hebron 119, 08035 Barcelona, Spain; 5 Department of Microbiology, Autonomous University of Barcelona, 08193 Bellaterra (Cerdanyola del Vallès), Spain; 6 Division of Allergy and Clinical Immunology, Brigham and Women’s Hospital, Boston, MA 02115, USA; 7 Harvard Medical School, Boston, MA 02115, USA

Respiratory viruses are a common cause of hospital admissions during the winter season worldwide. In addition to well-known viruses, recent hantavirus and Ebola outbreaks, which present the same initial non-specific symptoms as other viral infections, have raised interest in virology. Cytokine storm syndrome is involved in the pathogenicity of respiratory viruses (such as coronaviruses and influenza) as well as in many other viruses, such as Ebola, HIV, dengue, Zika, West Nile virus, hepatitis, enterovirus [1] and hantavirus [2]. Self-diagnosis tests for respiratory viruses are now available to the general population. The rate of false negatives in viral tests might be lower if the testing swab is inserted perpendicularly into the lower meatus of the nasal cavity, avoiding injury to the roof of the cavity. The technique is safer as non-perpendicular swab insertions have been linked to cerebrospinal fluid rhinorrhea and meningitis [3].

There is a paucity of treatments for respiratory viruses. Oseltamivir can reduce the duration of the symptomatic period by 25–30% in influenza-infected patients [4]. Recently, nirmatrelvir/ritonavir has been approved for the treatment of confirmed coronavirus cases [5]. Both treatments have been proven effective if administered within the first days of the symptomatic period, but their high cost and side effects limit their generalized use.

During the COVID-19 pandemic, early studies on drug repurposing at the beginning of the pandemic identified 28 compounds approved by the Food and Drug Administration, after studying the SARS-CoV-2 protein interaction map. Antihistamines were among them [6], and a role in modulating interleukins responses has been proposed [7]. Famotidine has been proven effective in randomized controlled trials for symptomatic treatment [8,9]. Epidemiological studies, involving tens of thousands of individuals, detected a reduction in COVID-19 cases in patients already taking antihistamines [10]. Other publications also associated its chronic prescription with a reduction in hospital admissions and deaths [11]. SARS-CoV-2 infection, which features intercurrent reactivations with new strains, may be associated with the recently described increase in thrombosis, although antihistamine-treated patients have shown lower rates of such cardiovascular events [12]. The classically described antihistamine interaction with the platelet-activating factor could explain the finding [13]. These drugs, due to their low side effect profile, are already included in numerous over-the-counter preparations available in pharmacies.

Prophylactic experience with antihistamines has also been described as all geriatric patients in nursing homes in Yepes (Toledo, Spain) were treated with them during the first COVID-19 wave. Symptomatic patients were additionally treated with azithromycin, an antibiotic that also has immunomodulatory activity beyond its antibacterial function [14,15,16]. When deaths in Spanish nursing homes were reaching more than 40% [17], none of those 84 elderly patients in Yepes required hospital admission or died, even though serological tests after the first wave confirmed that all had been infected [18]. Early treatment of 468 primary care patients diagnosed with COVID-19 in the same area reduced hospital admissions by half compared to rates in similar populations in the country [19]. Prophylactic use of azelastine nasal spray has also reduced the incidence of SARS-CoV-2 infections by more than half in a recent randomized controlled trial [20].

The lethal effects of hantavirus infection have been linked to an excessive immune response and are often treated with corticosteroids [21,22,23,24]. The incubation period may last up to 39 days [25]. Cardiopulmonary complications, similar to those observed in coronavirus infections and other respiratory viral diseases, are predominant in the Andean strain [26], which is the only hantavirus strain known to be transmissible between humans. In contrast, hemorrhagic fever with renal syndrome predominates in Asian variants [27], in which impaired platelet function may also increase thrombotic risk [28].

It remains uncertain whether antihistamines could play a role in reducing the progression of hantavirus infection, as no published evidence currently supports their use. However, the favorable safety profile of these drugs may facilitate future clinical trials or pragmatic clinical use. Further prospective studies are needed to confirm the prophylactic and therapeutic efficacy of antihistamines in specific viral infections involving cytokine storm syndromes, particularly when no specific treatments or vaccines are available.

Further research on respiratory viruses is needed, and contributions to *Viruses* and to this Special Issue are welcome.
